# Detection of Prostatic Inflammation From Peripheral Lymphocyte Count and Free/Total PSA Ratio in Men With LUTS/BPH

**DOI:** 10.3389/fphar.2020.00589

**Published:** 2020-04-30

**Authors:** Xinyang Liao, Zhuang Tang, Jianzhong Ai, Hang Xu, Shiyu Zhang, Liangren Liu, Shi Qiu, Ping Tan, Yu Fan, Lu Yang, Qiang Wei

**Affiliations:** ^1^Department of Urology, Institute of Urology, West China Hospital, Sichuan University, Chengdu, China; ^2^Department of Urology, Sun Yat-sen Memorial Hospital, Guangzhou, China; ^3^Center of Biomedical Big Data, West China Hospital, Sichuan University, Chengdu, China

**Keywords:** lower urinary tract symptoms, prostatic hyperplasia, prostate-specific antigen, inflammation, prostatitis

## Abstract

**Objective:**

Identifying biomarkers of prostatic inflammation has been a question of great interest in the development of anti-inflammatory pharmacotherapy for lower urinary tract symptoms suggestive of benign prostatic hyperplasia (LUTS/BPH). Systemic inflammation and serum prostate-specific antigen (PSA) have been linked with prostatic inflammation. This study set out to develop a diagnostic model for prostatic inflammation using clinical and laboratory parameters.

**Methods:**

We included LUTS/BPH patients undergoing transurethral resection of the prostate. The severity of prostatic inflammation was determined by pathological review. Clinical manifestations and preoperative laboratory test results were recorded. We used LASSO regression with 10-fold cross-validation to select variables with the most diagnostic value of prostatic inflammation. Furthermore, we used multivariable logistic regression analysis to develop the diagnostic model, presented in a nomogram. The discrimination, calibration of the post-LASSO diagnostic model, and the model supplemented with clinical parameters were assessed. Decision curve analysis was performed.

**Results:**

A total of 164 patients were included. Of all patients, 97 (59.1%) had no or mild prostatic inflammation, and 67 (40.9%) had moderate to severe prostatic inflammation. A higher peripheral white blood cell count, higher peripheral lymphocyte count, lower free/total (f/t) PSA ratio, and acute urinary retention history were associated with a higher risk of moderate to severe prostatic inflammation. Peripheral lymphocyte count and f/t PSA ratio were selected by the LASSO method and entered into the nomogram. The post-LASSO diagnostic model had an AUC of 0.756 (95% CI: 0.684–0.829) and good calibration. The addition of clinical parameters failed to show incremental diagnostic value. The decision curve analysis demonstrated that the post-LASSO laboratory nomogram was clinically useful.

**Conclusion:**

Our findings demonstrated that peripheral lymphocyte count and f/t PSA ratio appear to be reliable diagnostic markers, based on which we build a clinically useful nomogram for prostatic inflammation. This diagnostic model could facilitate the development of anti-inflammatory pharmacotherapy for LUTS/BPH. Before this model is adopted in clinical practice, future validation is needed to determine its clinical utility.

## Introduction

Prostatic inflammation plays a crucial role in the pathogenesis and progression of lower urinary tract symptoms secondary to benign prostatic hyperplasia (LUTS/BPH). It is now well established from a variety of studies that prostatic inflammation is associated with higher International Prostate Symptom Score (IPSS) and prostate volume. (Di Silverio et al., 2003; Nickel et al., 2008; Robert et al., 2009) The inflammatory cells in prostates can give rise to cytokines and growth factors that stimulate the hyper-proliferation of prostatic stromal and epithelial cells. Besides, it has been hypothesized that inflammatory infiltrate could lead to tissue damage, a continuous process of wound healing, and subsequently prostatic enlargement (De Nunzio et al., 2011).

Prostatic inflammation is also demonstrated to be a treatment target for male LUTS/BPH and predictive of treatment response. Previous studies (Addla et al., 2006; Altavilla et al., 2012) have established that anti-inflammatory drugs are effective in the treatment of LUTS/BPH. And a recent meta-analysis of randomized controlled trials (Kahokehr et al., 2013) concluded that non-steroidal anti-inflammatory drugs improved urinary symptom and flow measures. Furthermore, previous research (Kwon et al., 2010) found that a higher grade of prostatic inflammation portends poorer response to medical treatment, including alpha-adrenergic blockers and 5-alpha reductase inhibitors. BPH patients with prostatic inflammation might benefit from anti-inflammatory pharmacotherapy, whereas those without prostatic inflammation might not.

How to identify patients with prostatic inflammation is of interest because of the pivotal role of prostatic inflammation in pathogenesis and management of LUTS/BPH. However, it is a challenging clinical problem. Prostatic inflammation can be pathologically confirmed in patients undergoing prostate biopsies. But the majority of patients bothered by LUTS/BPH do not receive any prostate biopsy. Previous studies explored laboratory biomarkers, including interleukin 8 in seminal plasma (Liu et al., 2009) and inducible T-cell co-stimulator in urine (Robert et al., 2011). These biomarkers can help identify prostatic inflammation. However, neither of these tests are available in clinical practice and can be expensive.

Systemic inflammation contributes to the initiation or progression of prostatic inflammation. Previous research has established that systemic inflammatory markers, including C-reactive protein, soluble tumor necrosis factor-alpha receptor II, interleukin 6 (Schenk et al., 2010), neutrophil-lymphocyte ratio (Ozer et al., 2017), and peripheral white blood cell (WBC) count (Fujita et al., 2014) are correlated with the severity of LUTS/BPH. Besides, some risk factors of LUTS/BPH, including obesity (Parsons et al., 2006), atherosclerosis (Berger et al., 2006), periodontal disease (Wu et al., 2019), and diabetes (Breyer and Sarma, 2014) are associated with systemic inflammation. Taken together, these studies suggest that systemic inflammation may worsen BPH symptoms by inducing prostatic inflammation.

Systemic inflammatory markers obtained from routine admission blood tests can be indicators of systemic inflammation. In addition to systemic inflammation, free/total prostate-specific antigen (f/t PSA) ratio (Jung et al., 1998) and clinical symptoms (Nickel et al., 2008) were also reported to be correlated with prostatic inflammation. This study set out to develop a diagnostic model for prostatic inflammation using clinical and laboratory parameters.

## Methods

### Cohort

**Figure 1:**
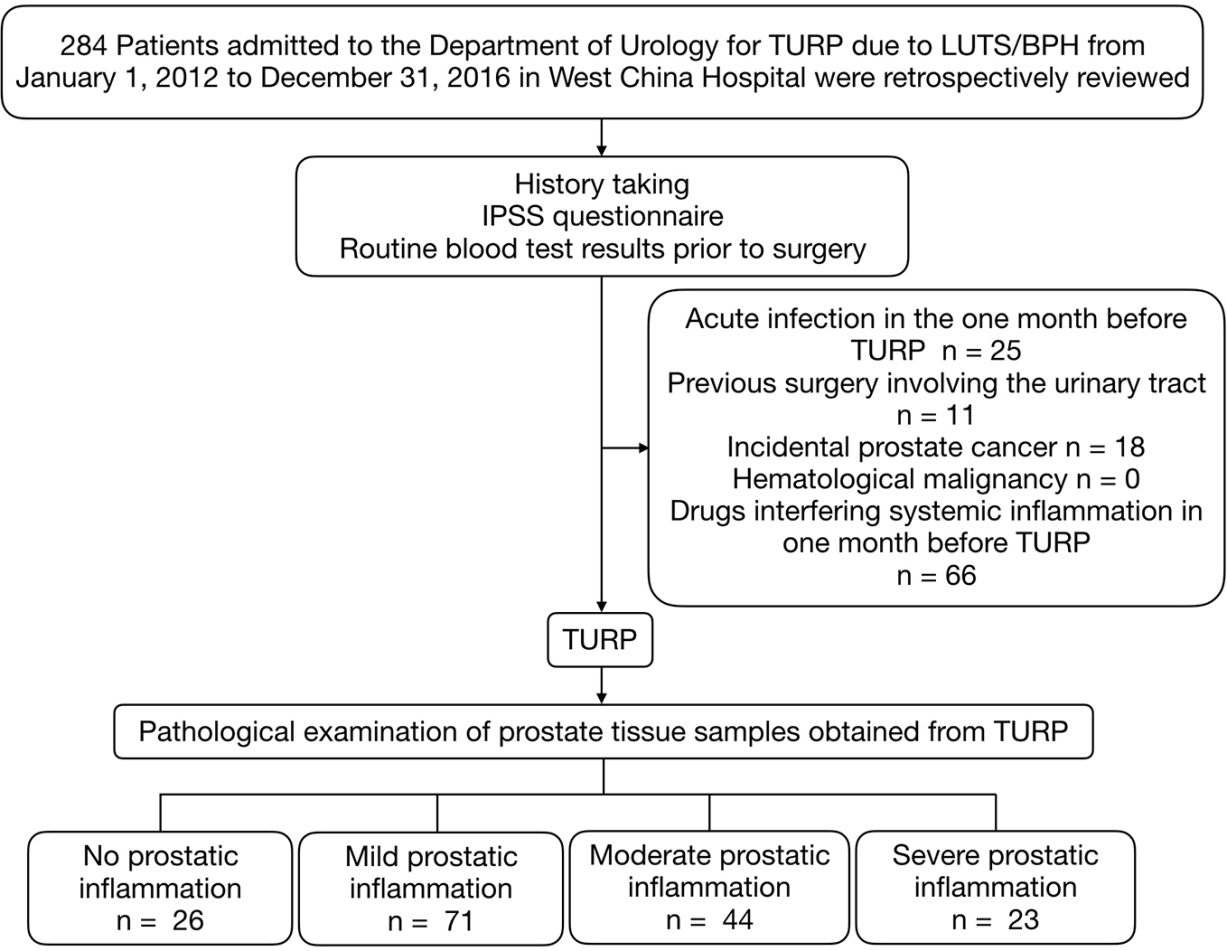
Patients selection process. TURP, transurethral resection of the prostate; IPSS, International Prostate Symptom Score; LUTS/BPH, lower urinary tract symptoms secondary to benign prostatic hyperplasia.

This study was retrospectively carried out at the Department of Urology in West China Hospital from January 1, 2012 to December 31, 2016. All data and specimens were collected anonymously without any identifier. We evaluated 284 male patients who received the transurethral resection of the prostate (TURP) due to LUTS/BPH. Exclusion criteria were any acute infection in one month before TURP, previous surgeries involving the urinary tract, incidental prostate cancer, hematological malignancy or the use of medicine interfering with the peripheral blood parameters in the three months before surgery, which included non-steroidal anti-inflammatory drugs, phosphodiesterase type 5 inhibitors, vitamins, and statin (**Figure 1**).

### Variates

**Figure 2:**
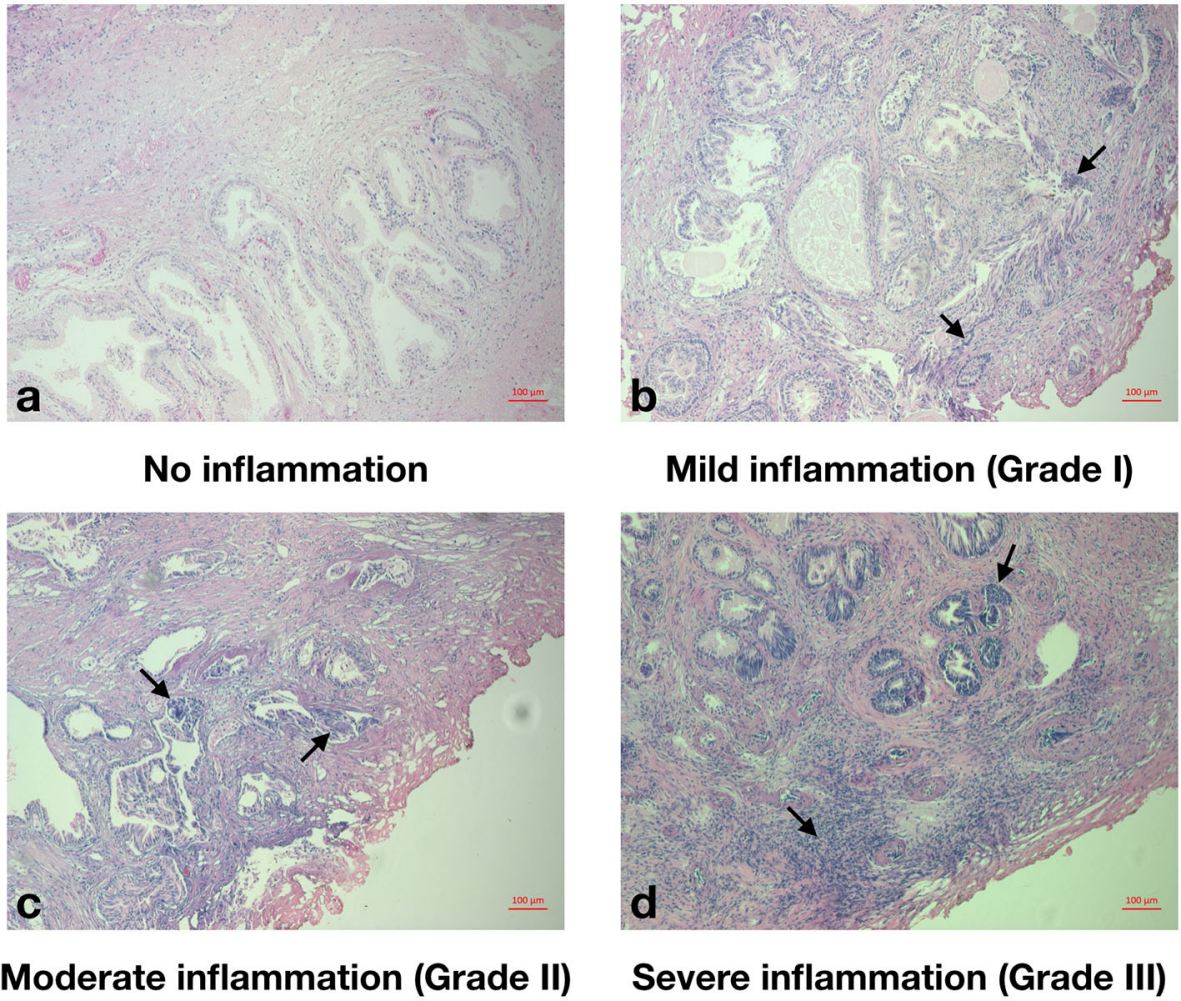
Histopathological grade of prostatic inflammation. **(A)**, No prostatic inflammation. There was no inflammatory cell. **(B)**, Mild prostatic inflammation (Grade I). There were scattered inflammatory cells infiltrate within the stroma. **(C)**, Moderate prostatic inflammation (Grade II). There were non-confluent lymphoid nodules. **(D)**, Severe prostatic inflammation (Grade III). There were large inflammatory areas with confluence of infiltrate.

Free and total PSA, IPSS, acute urinary retention (AUR) history, routine admission blood test results including peripheral WBC count, neutrophil count, lymphocyte count, platelet, albumin, fibrinogen, neutrophil-lymphocyte ratio (NLR), platelet to lymphocyte ratio (PLR), and systemic immune-inflammation index (SII) were recorded within one week before surgery. Total and free PSA were measured *via* automated electrochemiluminescent immunoassays using the Elecsys assay kits from Roche Diagnostics. Prostate volumes (PV) were assessed by transrectal ultrasound, using the Philips HDI 5000 ultrasound system and the standard ellipsoid formula (width × height × length × π/6) as per Rodriguez et al. (2008). IPSS was categorized as “asymptomatic” (0), “mildly symptomatic” (1–7), “moderately symptomatic” (8–19), and “severely symptomatic” (20–35). Prostatic inflammation of TURP specimen was individually graded by XL and ZT, according to the criteria recommended by North American Chronic Prostatitis Collaborative Research Network (CPCRN) and International Prostatitis Collaborative Network (IPCN) (Nickel et al., 2001) (**Figure 2**). Divergences were resolved by QW.

### Statistical Analysis

Categorical variables were described by frequencies and percentages. Continuous variables were described by means and standard deviations. We compared patient characteristics of two groups (no or mild prostatic inflammation group vs. moderate or severe prostatic inflammation group) using the Student's t-test for continuous variables and the Chi-squared test for categorical variables. The univariate logistic regression model was used to evaluate the associations between patient characteristics and the grade of prostatic inflammation.

By the least absolute shrinkage and selection operator (LASSO) method with 10-fold cross-validation, the optimal tuning parameter lambda (λ) was chosen as the highest λ for which the mean-squared error was within one standard deviation of the minimum (Hastie et al., 2009). With the optimal λ identified, variables with non-zero coefficients were the ones with most diagnostic value, thus selected into the diagnostic nomogram. We used a multivariable binary logistic regression model of selected variables to develop the nomogram. We assessed the model discrimination by the receiver-operating characteristic (ROC) curve and reported the area under the curve (AUC). We determined the optimal cutoff by Youden's index and calculated the sensitivity and specificity. AUCs of the post-LASSO model and the model supplemented by clinical parameters were compared. The calibration curve with the bootstrap approach (the number of bootstrap repetitions B = 500) was plotted to assess the calibration of the nomogram, accompanied by the Hosmer-Lemeshow test in which a signiﬁcant p-value indicates the model doesn't calibrate perfectly. Decision curve analysis was conducted. We conducted all the analyses using R software version 3.4.1 (http://www.r-project.org). Statistical significance was defined as a two-tailed p-value <0.05. The data collected and analyzed in this study is publicly available from Figshare (DOI: 10.6084/m9.figshare.10033253.v1). The R script of data analysis was available as **Supplementary Material**.

## Results

### Patient Characteristics

A total of 164 LUTS/BPH patients who underwent TURP with a mean age of 69.9 years were included in the study. Of all patients, 26 patients (15.9%) had no prostatic inflammation, while 71 (43.3%) patients had mild, 44 (26.8%) had moderate, and 23 (14.0%) had severe prostatic inflammation (**Table 1**). In the entire cohort of LUTS/BPH patients, 9 (5.49%) individuals were mildly symptomatic, 83 (50.61%) individuals were moderately symptomatic, and 72 (43.90%) individuals were severely symptomatic. Patients with moderate to severe prostatic inflammation had higher peripheral WBC counts, higher peripheral lymphocyte counts, lower f/t PSA ratios and lower NLRs, and were more likely to have a history of AUR (p < 0.05).

**Table 1:** Baseline characteristics of patients.

Grade of prostatic inflammation	0-1 (No or mild inflammation)	1-2 (Moderate or severe inflammation)	P-value
**Number of patients**	26 (no) + 71 (mild)	44 (moderate) + 23 (severe)	
**Age, y**	70.27 ± 7.25	69.48 ± 7.77	0.506
**Prostate volume, cm^3^**	47.57 ± 20.78	54.02 ± 22.20	0.059
**IPSS (continuous)**	18.56 ± 7.40	19.85 ± 6.64	0.253
**IPSS (categorical)**			0.504
Mildly symptomatic	6 (6.19%)	3 (4.48%)	
Moderately symptomatic	52 (53.61%)	31 (46.27%)	
Severely symptomatic	39 (40.21%)	33 (49.25%)	
**Platelet, 10^3^/µL**	161.11 ± 67.50	173.24 ± 60.82	0.241
**WBC, 10^3^/µL**	5.84 ± 1.63	6.42 ± 1.74	0.030
**Neutrophil, 10^3^/µL**	3.78 ± 1.36	3.90 ± 1.28	0.544
**Lymphocyte, 10^3^/µL**	1.49 ± 0.51	1.87 ± 0.59	< 0.001
**Albumin, g/L**	40.71 ± 3.49	40.72 ± 3.46	0.987
**Fibrinogen, g/L**	2.99 ± 0.81	3.15 ± 0.86	0.234
**Total PSA, ng/ml**	5.60 ± 5.77	7.41 ± 7.66	0.086
**Free PSA, ng/ml**	1.25 ± 1.20	1.17 ± 1.06	0.682
**f/t PSA ratio, %**	26.25 ± 11.39	18.78 ± 8.68	< 0.001
**NLR**	2.82 ± 1.51	2.21 ± 0.79	< 0.001
**PLR**	117.04 ± 61.04	100.90 ± 46.82	0.070
**SII**	682.94 ± 467.95	632.35 ± 311.77	0.440
**A history of AUR**			0.044
None	82 (84.54%)	47 (70.15%)	
Yes	15 (15.46%)	20 (29.85%)	

IPSS, International Prostate Symptom Score; WBC, white blood cell; PSA, prostate-specific antigen; f/t PSA ratio, free to total prostate-specific antigen ratio; NLR, neutrophil lymphocyte ratio; PLR, platelet to lymphocyte ratio; SII, systemic immune-inflammation index; AUR, acute urinary retention.

### Univariate Analysis

In the univariate logistic analysis, associated with a higher risk of mild to severe prostatic inflammation were acute urinary retention history (OR = 1.84, 95% CI: 1.09–2.62, p = 0.03), a lower f/t PSA ratio (OR = 0.93, 95% CI: 0.89–0.96, p < 0.01), a higher peripheral lymphocyte count (OR = 2.26, 95% CI: 1.66–2.92, p < 0.01), a higher WBC count (OR = 1.21, 95% CI: 1.02–1.48, p = 0.03) and a lower NLR (OR = 0.53, 95% CI: 0.18–0.83, p < 0.01). And it's noteworthy that prostate volume was also correlated with a higher risk of moderate to severe prostatic inflammation and trended towards statistical significance (OR = 1.01, 95% CI: 1.00–1.03, p = 0.06) (**Table 2**).

**Table 2:** Univariate analysis on the associations of clinical and laboratory parameters with moderate to severe prostatic inflammation.

Variable	Mean + SD/ Frequency (Percentage)	OR (95% CI)	P value
**Age, y**	69.95 + 7.45	0.99 (0.95, 1.03)	0.50
**Prostate volume, cm^3^**	50.21 + 21.54	1.01 (1.00, 1.03)	0.06
**IPSS**	19.09 + 7.10	1.03 (0.98, 1.07)	0.25
**Total PSA, ng/ml**	6.34 + 6.64	1.04 (0.99, 1.09)	0.10
**Free PSA, ng/ml**	1.23 + 1.19	0.94 (0.65, 1.22)	0.86
**f/t PSA ratio (%)**	22.17 + 8.82	0.93 (0.89, 0.96)	<0.01
**Platelet, 10^3^/µL**	166.07 + 64.93	1.00 (1.00, 1.01)	0.24
**WBC, 10^3^/µL**	6.08 + 1.70	1.21 (1.02, 1.40)	0.03
**Neutrophil, 10^3^/µL**	3.83 + 1.33	1.07 (0.84, 1.31)	0.54
**Lymphocyte, 10^3^/µL**	1.65 + 0.57	2.26 (1.66, 2.92)	<0.01
**Albumin, g/L**	40.71 + 3.46	1.00 (0.91, 1.09)	0.99
**Fibrinogen, g/L**	3.05 + 0.83	1.23 (0.85, 1.61)	0.23
**NLR**	2.57 + 1.30	0.53 (0.18, 0.83)	<0.01
**PLR**	110.45 + 56.09	0.99 (0.99, 1.00)	0.08
**SII**	662.27 + 411.03	1.00 (1.00, 1.00)	0.44
**A history of AUR**			
**None**	129 (78.66%)	ref	
**Yes**	35 (21.34%)	1.84 (1.09, 2.62)	0.03

SD, Standard Deviation; IPSS, International Prostate Symptom Score; WBC, White Blood Cell; PSA, prostate-specific antigen; f/t PSA ratio, free to total prostate-specific antigen ratio; NLR, neutrophil lymphocyte ratio; PLR, platelet to lymphocyte ratio; SII, systemic immune-inflammation index; AUR, acute urinary retention.

### LASSO Regression and Diagnostic Model Development

**Figure 3:**
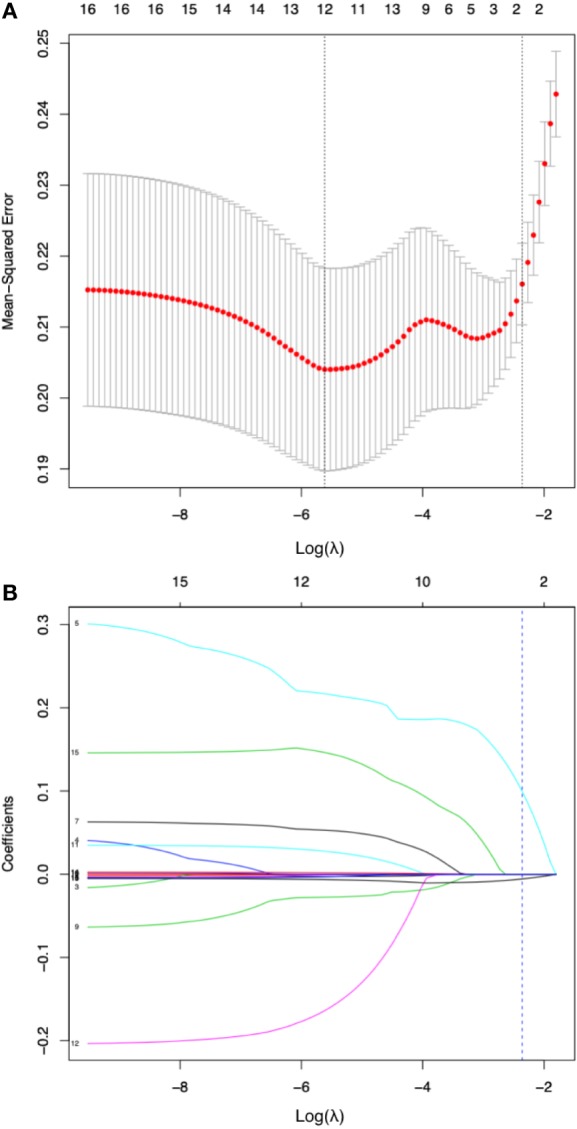
Diagnostic variable selection by the least absolute shrinkage and selection operator (LASSO) method. **(A)**.Tuning parameter lambda (λ) selection in the LASSO model with 10-fold cross validation and one-standard-error rule (the highest λ for which the mean-squared error was within one standard deviation of the minimum was chose as the optimal λ). The mean-squared error was plotted versus log(λ). Dotted vertical lines were drawn at the log (λ) with minimum mean-squared error (left one) or the optimal log(λ) by one-standard-error rule (right one). A λ value of 0.094 with log (λ) being -2.360 was chosen as optimal. **(B)** coefficient of the 16 clinical or laboratory parameters plotted against log(λ) sequence. Each curve corresponds to a variable; the dotted vertical blue line indicates the optimal value of log(λ) by one-standard-error rule at which two variables, the peripheral lymphocyte count and f/t PSA ratio had nonzero coefﬁcients and the most diagnostic value.

**Figure 4:**
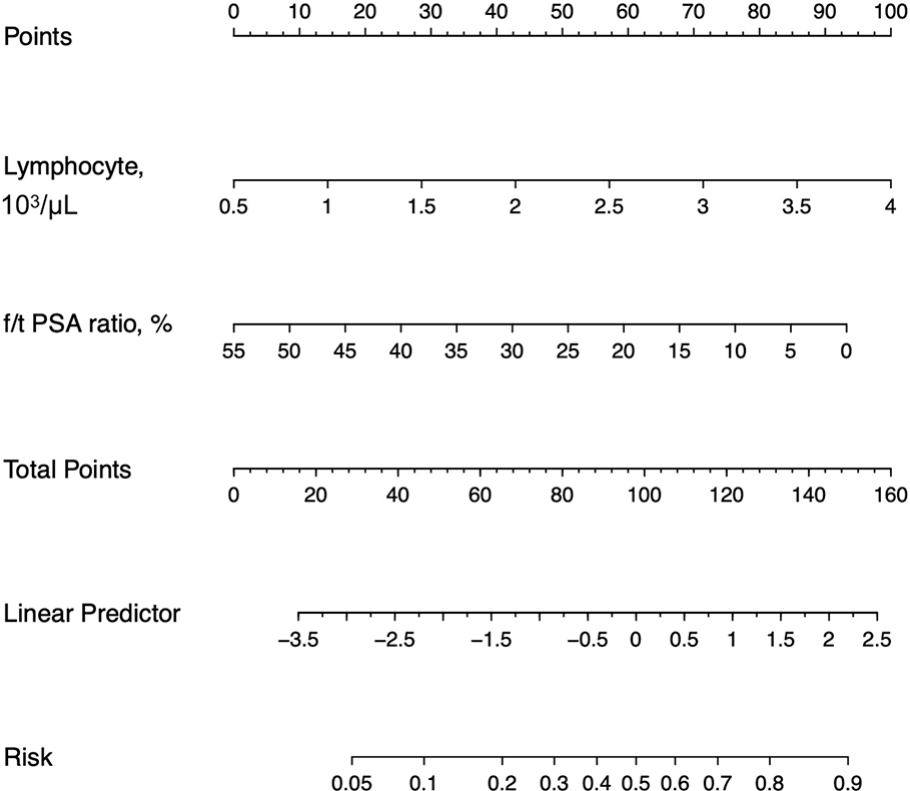
Nomograms for the laboratory model. f/t PSA ratio = free to total prostate-specific antigen ratio.

By LASSO regression with 10-fold cross-validation and the one-standard-error rule, the optimal λ was 0.094. Among all 16 variables, the peripheral lymphocyte count and f/t PSA ratio had nonzero coefﬁcients, thus selected in the final diagnostic model (**Figure 3**). The diagnostic model was built by multivariate logistic regression, and the nomogram of this model was shown in **Figure 4**.

### Discrimination, Calibration, and Decision Curve Analysis

**Figure 5:**
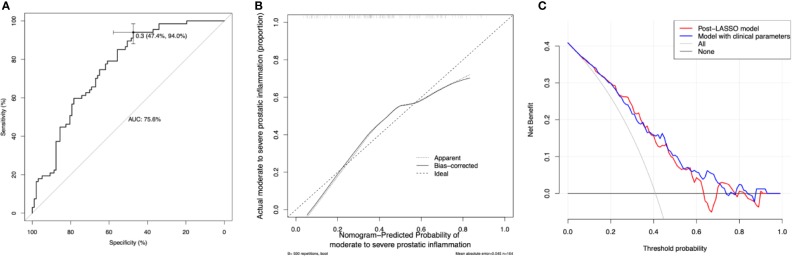
Model discrimination, calibration, and decision curve analysis. **(A)** Receiver operating characteristic curves (ROCs) for the diagnostic models. AUC = the area under the ROC curve. The laboratory model had an AUC of 0.756 (95% confidence interval 0.684–0.829) the optimal cutoff is 0.3, with the sensitivity of 0.94 and specificity of 0.47. **(B)** Calibration curve of the post-LASSO diagnostic nomogram accompanied by the bootstrap approach (the number of bootstrap repetitions B = 500). Calibration curve depicts the calibration of in terms of the agreement between the predicted risks of prostatic inflammation and observed prostatic inflammation. The y-axis represents the actual prostatic inflammation. The x-axis represents the predicted prostatic inflammation. The diagonal dashed line represents a perfect prediction by an ideal model. The dotted (apparent) and solid (bootstrap bias corrected) line represents the performance of the nomogram, of which a closer ﬁt to the diagonal dashed line represents a better prediction. **(C)** Decision curve analysis for the post-LASSO simpler nomogram and the model supplemented with clinical parameters. The y-axis measures the net beneﬁt. The x-axis is the risk threshold probability that changes from 0 to 1. The red line represents the post-LASSO nomogram. The blue line represents the model with clinical parameters. The grey line represents the assumption that all patients have prostatic inflammation. The black line represents the assumption that no patients have prostatic inflammation. The decision curve analysis yielded a range of thresholds (8 to 63%) at which using the post-LASSO model to diagnose prostatic inflammation adds more beneﬁt than the treat-all-patients scheme or the treat-none scheme.

The discrimination of the post-LASSO simpler model and the model supplemented by clinical parameters was accessed by the AUC of ROC curves (**Figure 5A**). The post-LASSO model had an AUC of 0.76 (0.68–0.83), while the model supplemented with clinical parameters including prostate volume and history of AUR, and IPSS had an AUC of 0.77 (0.70–0.84) (**Table 3**). The calibration curve of the post-LASSO nomogram for prostatic inflammation demonstrated good agreement between prediction and observation (**Figure 5B**). The Hosmer-Lemeshow test yielded a nonsigniﬁcant statistic (P = 0.432). The decision curve analysis of the post-LASSO model and the one supplemented with clinical parameters were presented in **Figure 5C**. The decision curve showed that if the threshold probability is between 8 to 63%, using the laboratory test nomogram to predict prostatic inflammation brings more beneﬁt than either the treat-all-patients scheme or the treat-none scheme. Within this range, two model lines overlap each other, which suggest net beneﬁt was comparable between the post-LASSO laboratory test nomogram and the model with clinical parameters.

**Table 3:** Risk factors for prostatic inflammation in patients with LUTS/BPH.

Intercept and Variable	Post-LASSO model			Model supplemented with clinical parameters		
	Beta	OR (95% CI)	P	Beta	OR (95% CI)	P
**Intercept**	-0.808	0.446 (0.110, 1.760)	0.251	-1.106	0.331 (0.048, 2.154)	0.25
**Lymphocyte, 10^3^/μL**	1.216	3.374 (1.784, 6.768)	<0.01	1.207	3.342 (1.758, 6.735)	<0.01
**f/t PSA ratio, %**	-0.072	0.930 (0.894, 0.964)	<0.01	-0.070	0.932 (0.894, 0.968)	<0.01
**Prostate volume**	NA	NA	NA	0.008	1.008 (0.991, 1.026)	0.35
**A history of AUR**	NA	NA	NA	0.706	2.026 (0.821, 5.103)	0.13
**IPSS**	NA	NA	NA	-0.015	0.985 (0.931, 1.040)	0.58
**AUC of ROC curves**		0.756 (0.684-0.829)			0.769 (0.698-0.840)	

Beta is the regression coefﬁcient.

LUTS/BPH, lower urinary tract symptoms secondary to benign prostatic hyperplasia; LASSO, The least absolute shrinkage and selection operator; OR, Odds ratio; CI, Confidence interval; f/t PSA ratio, free/total prostate-specific antigen; AUR, acute urinary retention; IPSS, International Prostate Symptom Score; AUC, area under the curve; ROC curves, receiver-operating characteristic curves; NA, not available.

## Discussion

In this study, we found that f/t PSA ratio and peripheral WBC count had great diagnostic value for prostatic inflammation. And we developed a new diagnostic model for prostatic inflammation in LUTS/BPH based on these two variables. The diagnostic model had good discrimination, calibration, and clinical benefit. The addition of other clinical parameters failed to bring incremental diagnostic value.

It's noteworthy that this study was conducted solely for LUTS/BPH patients, and we even excluded patients with incidental prostate cancer to reduce bias. Therefore, we do not know whether this model would apply to cancer patients. Using our model to identify prostatic inflammation would be useful for the management and pharmacotherapy development of LUT/BPH due to the strong association between prostatic inflammation and LUTS/BPH. (Zlotta et al., 2014) But whether prostatic inflammation is correlated with prostate cancer is still a question of debate (Langston et al., 2019).

Interestingly, a lower f/t PSA ratio was associated with a higher risk of more severe prostatic inflammation, and this finding broadly supports the work of Jung's (Jung et al., 1998), which also linked a lower f/t PSA ratio to chronic inflammation of the prostate. A possible explanation for this might be that the production of alpha 1-antichymotrypsin, with which free PSA predominately forms the complex, increases not only in cancer cells but also in hyperplastic cells under inflammatory conditions.

In our study, we found that peripheral WBC count is associated with prostatic inflammation. This finding corroborates the findings of the previous work by Dr. Kazutoshi et al. (Fujita et al., 2014), which demonstrated that peripheral WBC count was associated with the severity of LUTS/BPH. It can thus be suggested that a higher peripheral WBC count is representative of a higher level of systemic inflammatory status, which exacerbates prostatic inflammation and then aggravates LUTS/BPH. Besides, we found that the peripheral lymphocyte count was positively associated with prostatic inflammation, confirming that systemic inflammation and prostatic inflammation are intrinsically correlated.

To our best knowledge, this is the first attempt to develop a diagnostic model for prostatic inflammation using the routine blood test and the PSA test. And our model proved to be clinically useful. However, our study had several limitations. First, our sample size was relatively small due to the stringent eligibility criteria. A larger cohort will be needed to validate our diagnostic model further. Second, we didn't measure the serum levels of interleukins, knowing it can be expensive and unlikely to be integrated into daily practice due to the high cost. At last, we didn't collect urinalysis data, which could be useful to describe our study population.

Despite these limitations, we developed a diagnostic model for prostatic inflammation, which is ready to use and proved to be clinically useful. And our findings demonstrated that peripheral lymphocyte count and f/t PSA ratio appear to be reliable diagnostic markers for prostatic inflammation, suggesting prostatic inflammation is correlated with systemic inflammation and might affect the PSA level.

## Conclusion

This study is the first attempt to develop a diagnostic model for prostatic inflammation using the routine blood test and the PSA test. Our findings demonstrated that peripheral lymphocyte count and f/t PSA ratio appear to be reliable diagnostic markers, based on which we build a clinically useful nomogram for prostatic inflammation. This diagnostic model could facilitate the development of anti-inflammatory pharmacotherapy for LUTS/BPH. Before this model is adopted in clinical practice, future validation is needed to determine its clinical utility.

## Data Availability Statement

The data collected and analyzed in this study is publicly available from Figshare (DOI: 10.6084/m9.figshare.10033253.v1).

## Ethics Statement

Ethical review and approval were not required for the study on human participants in accordance with the local legislation and institutional requirements. Written informed consent for participation was not required for this study in accordance with the national legislation and the institutional requirements.

## Author Contributions

XL contributed to analysis and interpretation of data and drafting of the manuscript. ZT contributed to acquisition of data. JA contributed to interpretation of data. HX and SZ contributed to statistical analysis. LL, SQ, YF and PT contributed to critical revision of the manuscript. LY and QW contributed to conception, design, supervision, and obtaining funding.

## Funding

This research was funded by the National key research and development program of China (Grant No.SQ2017YFSF090096), the Prostate Cancer Foundation Young Investigator Award 2013, the National Natural Science Foundation of China (Grant No. 81300627, 81370855, 81702536, 81770756), Programs from Science and Technology Department of Sichuan Province (Grant No. 2014JY0219 and 2017HH0063), Young Investigator Award of Sichuan University 2017, and a grant from 1.3.5 project for disciplines of excellence, West China Hospital, Sichuan University (ZYGD18011).

## Conflict of Interest

The authors declare that the research was conducted in the absence of any commercial or financial relationships that could be construed as a potential conflict of interest.
