# Supplementary material for: Detection of Prostatic Inflammation From Peripheral Lymphocyte Count and Free/Total PSA Ratio in Men With LUTS/BPH
Source: Front Pharmacol. 2020 Apr 30;11:589. doi: 10.3389/fphar.2020.00589 (PMC7204507; doi:10.3389/fphar.2020.00589)
Supplement: Supplementary file 1 [file Table_1.docx]

library(rms)

library(pROC)

library(tidyverse)

library(dplyr)

library(plyr)

library(readr)

library(glmnet)

library(xlsx)

library(furniture)

library(ResourceSelection)

mydata <- read_csv("data of systemic and prostatic inflammation.csv")

#use IPSS as a categorical variable

mydata$x22 <- case_when(

between(mydata$x19,1,7) == TRUE ~ 0,

between(mydata$x19,8,19) == TRUE ~ 1,

between(mydata$x19,20,35) == TRUE ~ 2,

)

library("Hmisc")

label(mydata$x2) <- "Age, years"

label(mydata$x5) <- "Platelet, 10^3/uL"

label(mydata$x6) <- "WBC, 10^3/uL"

label(mydata$x7) <- "Neutrophil, 10^3/uL"

label(mydata$x8) <- "Lymphocyte, 10^3/uL"

label(mydata$x9) <- "Albumin, g/L"

label(mydata$x10) <- "Fibrinogen, g/L"

label(mydata$x11) <- "SII"

label(mydata$x12) <- "NLR"

label(mydata$x13) <- "PLR"

label(mydata$x14) <- "Total PSA, ng/ml"

label(mydata$x15) <- "Free PSA, ng/ml"

label(mydata$x16) <- "f/t PSA ratio, %"

label(mydata$x17) <- "Prostate volume, cm^3"

mydata$x18 <- factor(mydata$x18, levels = c(0,1), labels=c("None","Yes"))

label(mydata$x18) <- "Acute urinary retention history"

label(mydata$x19) <- "IPSS"

mydata$x21 <- factor(mydata$x21, levels = c(0,1), labels=c("No or mild inflammation","Moderate or severe inflammation"))

label(mydata$x21) <- "Inflammation grade of prostate"

label(mydata$x22) <- 'IPSS severity'

mydata$x22 <- factor(mydata$x22, levels = c(0,1,2), labels=c("Mildly symptomatic","Moderately symptomatic","Severely symptomatic"))

#lasso regression

independent <- model.matrix(x21~., mydata)[,-1]

dependent <- mydata$x21

set.seed(123)

cv.lasso <- cv.glmnet(independent, dependent, alpha = 1)

plot(cv.lasso)

dev.print(pdf, file="LASSO coefﬁcient proﬁles.pdf")

dev.off

fit = glmnet(independent, dependent, alpha=1)

print(fit)

plot(fit,xvar = 'lambda',label=TRUE)

#abline(v = log(cv.lasso$lambda.min), col = "red", lty = "dashed")

abline(v = log(cv.lasso$lambda.1se), col = "blue", lty = "dashed")

dev.print(pdf, file="Lambda selection.pdf")

dev.off

cv.lasso$lambda.1se

coef(cv.lasso, cv.lasso$lambda.1se)

#table1

table1(mydata,

x2, x5, x6, x7, x8, x9, x10, x11, x12, x13, x14, x15, x16, x17, x18, x19,x22,

splitby = ~x21, digits = 2, rounding_perc = 2,

test = TRUE, export = "table1")

#univariate analysis

covariates <- colnames((mydata)[,-ncol(mydata)])

uni_formulas <- sapply(covariates,

function(x) as.formula(paste('x21~', x)))

uni_formulas

uni_models <- lapply(uni_formulas, function(x){glm(x, data = mydata,family = binomial())})

uni_models

uni_results <- lapply(uni_models, function(x) {

x <- summary(x)

coef <- x$coefficients[2,]

return(coef)

})

class(uni_results)

str(uni_results)

res <- t(as.data.frame(uni_results, check.names = FALSE))

res

uni_conf <- lapply(uni_models, function(x) {

x <- confint(x)

conf <- x[2,]

return(conf)

})

str(uni_conf)

conf_total <- t(as.data.frame(uni_conf, check.names = FALSE))

conf_total

uni_final <- cbind(res,conf_total)

class(uni_final)

uni_final[,1] <- exp(uni_final[,1])

uni_final[,5] <- exp(uni_final[,5])

uni_final[,6] <- exp(uni_final[,6])

uni_final <- as.data.frame(uni_final)

uni_final <- round(uni_final,2)

uni_final <- uni_final %>% mutate(OR=str_c(Estimate," (",`2.5 %`,", ", `97.5 %`, ") "))

uni_final

write.xlsx(uni_final, "uni_variate1.xlsx")

attach(mydata)

multi3 <- glm(x21~x8+x16, data = mydata,family = binomial())

sum_multi3 <- summary(multi3)

sum_multi3 <- sum_multi3$coefficients

conf_multi3 <- confint(multi3)

res_multi3 <- cbind(sum_multi3,conf_multi3)

res_multi3[,1] <- exp(res_multi3[,1])

res_multi3[,5] <- exp(res_multi3[,5])

res_multi3[,6] <- exp(res_multi3[,6])

res_multi3 <- as.data.frame(res_multi3)

res_multi3 <- round(res_multi3,2)

res_multi3 <- res_multi3 %>% mutate(OR=str_c(Estimate," (",`2.5 %`,", ", `97.5 %`, ") "))

res_multi3

write.xlsx(res_multi, "multi_variate3.xlsx")

multi4 <- glm(x21~x8+x16+x17+x18+x19, data = mydata,family = binomial())

sum_multi4 <- summary(multi4)

sum_multi4 <- sum_multi4$coefficients

conf_multi4 <- confint(multi4)

res_multi4 <- cbind(sum_multi4,conf_multi4)

res_multi4[,1] <- exp(res_multi4[,1])

res_multi4[,5] <- exp(res_multi4[,5])

res_multi4[,6] <- exp(res_multi4[,6])

res_multi4 <- as.data.frame(res_multi4)

res_multi4 <- round(res_multi4,2)

res_multi4 <- res_multi4 %>% mutate(OR=str_c(Estimate," (",`2.5 %`,", ", `97.5 %`, ") "))

res_multi4

write.xlsx(res_multi4, "multi_variate4.xlsx")

#nomogram

dd=datadist(mydata)

options(datadist="dd")

fit1<- lrm(x21~x8+x16, data = mydata)

fit1

sum.surv<-summary(fit1)

sum.surv

fit2<- glm(x21~x8+x16, data = mydata, family = binomial)

fit2

pre2 <- predict(fit2,type="response")

plot.roc(mydata$x21, pre2,

main="ROC Curve", percent=TRUE,

print.auc=TRUE,

ci=TRUE, of="thresholds",

thresholds="best",

print.thres="best",

transpose = FALSE)

rocplot2 <- roc(mydata$x21,pre2)

ci.auc(rocplot2)

dev.print(pdf, file="ROC.pdf")

dev.off

fit3<- lrm(x21~ x8+x16+x17+x18+x19, data = mydata)

fit3

sum.surv<-summary(fit3)

sum.surv

fit4<- glm(x21~x8+x16+x17+x18+x19, data = mydata, family = binomial)

fit4

pre4 <- predict(fit4,type="response")

plot.roc(mydata$x21, pre4,

main="ROC Curve", percent=TRUE,

print.auc=TRUE,

ci=TRUE, of="thresholds",

thresholds="best",

print.thres="best",

transpose = FALSE)

rocplot4 <- roc(mydata$x21,pre4)

ci.auc(rocplot4)

#Hosmer-Lemeshow Goodness Of Fit (GOF) Test

hoslem.test(mydata$x21, fitted(fit2))

sum_model1 <- summary(fit2)

sum_model1 <- sum_model1$coefficients

conf_model1 <- confint(fit2)

res_model1 <- cbind(sum_model1,conf_model1)

res_model1 <- as.data.frame(res_model1)

res_model1$OR <- exp(res_model1$Estimate)

res_model1$OR_CI2.5 <- exp(res_model1$`2.5 %`)

res_model1$OR_CI97.5 <- exp(res_model1$`97.5 %`)

res_model1 <- round(res_model1, 3)

res_model1 <- res_model1 %>% mutate(OR_2.5_97.5_SI=str_c(OR," (",OR_CI2.5,", ", OR_CI97.5, ") "))

res_model1

write.xlsx(res_model1, "supplemented model1.xlsx")

sum_model2 <- summary(fit4)

sum_model2 <- sum_model2$coefficients

conf_model2 <- confint(fit4)

res_model2 <- cbind(sum_model2,conf_model2)

res_model2 <- as.data.frame(res_model2)

res_model2$OR <- exp(res_model2$Estimate)

res_model2$OR_CI2.5 <- exp(res_model2$`2.5 %`)

res_model2$OR_CI97.5 <- exp(res_model2$`97.5 %`)

res_model2 <- round(res_model2, 3)

res_model2 <- res_model2 %>% mutate(OR_2.5_97.5_SI=str_c(OR," (",OR_CI2.5,", ", OR_CI97.5, ") "))

res_model2

write.xlsx(res_model2, "supplemented model2.xlsx")

plot(nomogram(fit1,

fun=plogis,

fun.at=c(.001, .01, .05, seq(.1,.9, by=.1), .95, .99, .999),

lp=T,

maxscale=100,

funlabel="Risk")

)

dev.print(pdf, file="Nomogram.pdf")

dev.off

f <- lrm(x21 ~ x8 + x16, data = mydata)

pred.logit <- predict(f, mydata)

phat <- 1/(1+exp(-pred.logit))

val.prob.ci(phat, x21, cex=1)

library(PredictABEL)

pr.e <- predict(fit2,mydata,type = c("response"))

plotCalibration(data=mydata,

cOutcome=17,

predRisk=pr.e,

rangeaxis=c(0,1))

fit1<- lrm(x21~ x8+x16, x = T, y = T, data = mydata)

cal1 <- calibrate(fit1,X=T,Y=T, method='boot',m=16,B=500)

cal1

plot(cal1,lwd=2,lty=1,

cex.lab=1.2, cex.axis=1, cex.main=1.2, cex.sub=0.6,

xlim = c(0,1),ylim= c(0,1),

xlab=paste("Nomogram-Predicted Probability of", '\n', 'moderate to severe prostatic inflammation', separate = '') ,

ylab="Actual moderate to severe prostatic inflammation (proportion)",

col=c("#00468BFF","#ED0000FF","#42B540FF")

)

lines(cal1[,c(1:3)],

type ="o",

lwd = 1,

pch = 16,

col=c("#00468BFF"))

abline(0,1,lty = 3,

lwd = 2,

col = c("#224444")

)

dev.print(pdf, file="calibration.pdf")

dev.off

#decision_curve

source("dca.R")

attach(mydata)

mydata <- as.data.frame(mydata)

mydata$SI = predict(fit2, type="response")

mydata$SI_CM = predict(fit4, type="response")

dca(data=mydata, outcome="x21", predictors=c("SI","SI_CM"))

dev.print(pdf, file="DCA.pdf")

dev.off

dca(data=mydata, outcome="x21", predictors="model",probability = FALSE)

dca(yvar=x21, xmatrix=model, prob='N')

library(rmda)

simple<- decision_curve(x21~x8+x16,data= mydata,

family = binomial(link ='logit'),

thresholds= seq(0,1, by = 0.01),

confidence.intervals = 0.95,

study.design = 'cohort')

complex<- decision_curve(x21~x8+x16+x17+x18+x19,data= mydata,

family = binomial(link ='logit'),

thresholds= seq(0,1, by = 0.01),

confidence.intervals = 0.95,

study.design = 'cohort')

List<- list(simple,complex)

plot_decision_curve(List,

curve.names=c('Post-LASSO model','Model with clinical parameters'),

cost.benefit.axis =FALSE,col= c('red','blue'),

confidence.intervals=FALSE,

standardize = FALSE,

xlab = "Threshold probability")

dev.print(pdf, file="DCA new.pdf")

dev.off
